# Time to use large-scale biobank databases in health policy and public health research

**DOI:** 10.1093/haschl/qxaf158

**Published:** 2025-08-09

**Authors:** José A Pagán, Vivian Hsing-Chun Wang, Hannah Sur

**Affiliations:** Department of Public Health Policy and Management, School of Global Public Health, New York University, New York, NY 10003, United States; Center for Population and Health Services Research, Department of Foundations of Medicine, NYU Grossman Long Island School of Medicine, Mineola, NY 11501, United States; Department of Public Health Policy and Management, School of Global Public Health, New York University, New York, NY 10003, United States

**Keywords:** large-scale biobank databases, health policy, public health

## Abstract

Large-scale biobank databases link data from thousands of participants and multiple sources such as electronic health records, surveys, imaging, and biomarkers. These data infrastructure research initiatives have the potential to inform health care and public health solutions that can address the needs of different populations, facilitate cross-sector collaboration, and improve the overall responsiveness of health care and public health systems. Despite this potential, large-scale biobanks are severely underutilized to answer health policy and public health research questions. Part of the reason is that information available in these data sources is usually collected for purposes such as improving health care within an integrated health care delivery system or advancing precision medicine. Still, the depth and breadth of these data collection efforts around the world together with advances in data science and artificial intelligence provide new opportunities to answer important health policy and public health questions using large-scale biobank data.

Large-scale biobank databases have revolutionized biomedical research given their size (often hundreds of thousands of participants), geographic coverage, and inclusion of diverse populations.^1,2^ Many of these databases have the capacity to link data from electronic health records (EHR), surveys, imaging, biomarkers, and include backend database features that allow their linking to externally sourced information such as spatial socioeconomic and environmental data. The UK Biobank integrates data from half a million participants in the United Kingdom (UK) and has more than 35 000 registered researchers in 90 countries.^1^ A similar effort in the United States (US) is the *All of Us* Research Program, a study from the National Institutes of Health that seeks to integrate survey, EHR, physical measurement, mobility, and genomic data from 1 million participants.^2^

Table 1 describes 6 examples of major large-scale biobank databases around the world that include comprehensive data and are largely accessible to researchers—*All of Us* (US), UK Biobank, Taiwan Biobank, China Kadoorie Biobank, 45 and Up Study (New South Wales, Australia), and the Korea Biobank Project (South Korea). All these large-scale biobanks include EHR and genetic data (whole genome sequencing, genotyping, and pharmacogenomics), and some of them facilitate the study of populations underrepresented in biomedical research such as ethnic and racial minority groups (*All of Us*), include health insurance records (China Kadoorie Biobank), contain mobility data (*All of Us*, UK Biobank, Korea Biobank Project), and have long follow up periods (45 and Up Study).

**Table 1.** Key large-scale biobank databases around the world.

Biobank/hyperlink	Sample	Years	Geographic coverage	Self-reported survey data	EHR and insurance ecords	Mobility data	Genetic data
*All of Us* Research Program	1 million + (18 + years of age)	2017-present	US, including US territories	Demographics, lifestyle, medical history, family health history	Integrated with EHR data	Collected via wearable health tracking devices	Whole genome sequencing, genotyping, pharmacogenomics
UK Biobank	500 000 (40-69 years of age)	2006-present	UK	Lifestyle, diet, mental health, cognitive function	Integrated with National Health Service records	Accelerometer, limited time frame	Whole genome sequencing, genotyping, whole exome sequencing
Taiwan Biobank	200 000+ (20-70 years of age)	2012-Present	Taiwan	Lifestyle, medical history, physical activity, diet, smoking, alcohol	Integrated with Taiwan National Health Insurance database		Whole genome sequencing data, epigenomics, metabolomics
China Kadoorie Biobank	512 000+	2004-present	China	Demographics, lifestyle, medical history, medication use	Electronic linkage with the national health insurance system		Whole genome sequencing, genotyping
45 and Up Study	250 000+ (45 + years of age)	2006-present	New South Wales, Australia	Lifestyle, medical history, environmental factors	Integrated with clinical/hospital and pharmacy records, cancer registry		Genotyping, phenotyping
Korea Biobank Project	500 000+ (40-69 years of age)	2008-present	South Korea	Lifestyle, dietary habits, medical history, family history	Integrated with national health records	Limited, collected through physical activity surveys	Genotyping, planned whole genome sequencing, exome sequence

Given the comprehensiveness of data included in large-scale biobanks, these major data infrastructure research initiatives have the potential to inform public health solutions that can address the needs of different populations, facilitate cross-sector collaboration, and improve the overall responsiveness of public health systems.^3^ Large-scale biobanks can also facilitate precision public health given the intentional inclusion of different populations during the recruitment process.^4^

Although biomedical scientists have embraced the use of large-scale biobanks in recent years to advance genetic research, their use is more limited in health policy and public health research. For example, a PubMed citation search over the last 10 years of the term “health policy” retrieved 255 citations out of 15 722 citations that also included the 6 large-scale biobank databases mentioned above. A similar search using “genetic” instead of “health policy” results in 27 times more citations (ie, 6884 citations).

## Using biobank databases to address health policy and public health research questions

There are many important health policy and public health research questions that can be answered using large-scale biobank databases. For example, both modifiable (eg, hypertension, obesity) and nonmodifiable (eg, BRCA genes, Apolipoprotein E ɛ4 gene) factors are associated with prevalent diseases such as cancer, cardiovascular disease, and Alzheimer's disease.^5^ How do the interactions among different risk factors—both biological and social—impact the design of public health interventions and inform the prioritization of resources allocated to the population groups most in need? Screening for health-related social needs (HRSNs) in health care settings is now required by the largest health insurance payer, the Centers for Medicare & Medicaid Services, but implementing screening tools for HRSNs can be costly.^6^ Which populations in a given community/region or health system should be prioritized to achieve a high return on investment?

The Public Health 3.0 framework is a call to action to develop a public health data infrastructure in the US that is reliable, timely, and actionable for all key stakeholders involved in improving population health everywhere.^7^ With an enormous amount of data points collected by different entities (eg, hospitals, community-based organizations, county department of health) to improve population health, what is the most important information that is needed from different populations to inform policies and programs designed to benefit the most people?

Questions like these ones are currently partially answered using existing data collection efforts such as the National Health Interview Survey and the Behavioral Risk Factor Surveillance System. However, findings based on these databases often come with limitations in implications for population groups with small sample size, diversity of population reached, and the data breadth included to more fully address gaps in public health knowledge.

## Strengths of large-scale biobank databases

### Population sample size

Large-scale biobanks allow us to get around sample size constraints and may help us obtain estimates for indicators for which there is either fragmented data or, simply, no national data (eg, the percentage of adults with diabetes who do not have this health condition under control^8^; discrimination in medical care^9^). These indicators can also be estimated for population groups that are usually excluded or not reported in many studies given a small number of participants, specific geographic areas such as a county or state, and study participants across time. The multiple sources of data typically integrated in large-scale biobanks (eg, EHRs, self-reported surveys, biomarkers) can also facilitate data reliability assessments and the validation of estimates.

### Population diversity

Advancing health equity by developing policies and programs to support marginalized populations is an important goal but there is limited evidence of what works for groups experiencing health disparities.^10,11^  *All of Us*, for example, focuses recruitment efforts on the inclusion of populations underrepresented in biomedical research (defined by race/ethnicity, sexual orientation and gender identity, urban or rural residence, access to health services, age, household income, education, and disability).^2^ Over 80% of *All of Us* participants identify with at least one of these populations and, as such, data from the study can be used to better understand health disparities within and across these population groups.^9^ The data volume and geographic heterogeneity of *All of Us* could also serve as a source of information for public health practitioners who are interested in adapting evidence-based interventions for a given geographic area or population.^10^

### Data diversity

Large-scale biobanks could potentially be used to conduct public health monitoring where timely disease detection is critical.^12^ This requires a data infrastructure that enables the exchange and integration of different types of data from multiple public and private entities that support health (ie, health care systems, state governments, national public health agencies, and the general public). Building such infrastructure is a major undertaking; large-scale biobanks could serve as testing sites to develop models for a national and integrated health data infrastructure. Potential uses of this data infrastructure range from infectious disease monitoring to addressing chronic health conditions such as hypertension and diabetes.^12^

## Considerations when using large-scale biobank data

We have provided a few examples to demonstrate the utility of large-scale biobank databases for health policy and public health research. Other promising uses include identifying the needs of populations that do not utilize public health services or the health care delivery system, designing value-based initiatives that incorporate multiple data sources of quality indicators, and supporting population health management efforts that better integrate HRSNs. Despite these potential uses and benefits, there are several key issues that need to be considered when using large-scale biobank data. Most biobank studies rely on volunteers instead of participants selected though population-based sampling, which means that findings may not be nationally representative and may not reflect the true experience of the different population groups included in the study. This concern can be ameliorated with the use of data calibration strategies to adjust estimates and align them with known values obtained from trusted data sources such as well-designed national health surveys.^13^

While large-scale biobanks integrate data from multiple sources, consolidated databases may only capture a limited profile of study participants. Participation in biobank studies is also highly dependent on recruitment and retention strategies. Missing data may be a concern if participant engagement is low.

## Conclusion

With the increased use of artificial intelligence and machine learning in research, the use of massive health-related databases to generate new knowledge will only grow.^14^ Large-scale biobanks are at the forefront of biomedical research efforts—particularly in genetic research and precision medicine—but they are sharply underutilized to answer health policy and public health research questions. Scientists with an interest in addressing health policy and public health issues and challenges should explore different opportunities to answer important research questions with the best available data even though this information may have been collected largely to improve health care within an integrated health care delivery system or advancing precision medicine.^15^ Although the intended use of large-scale biobank data has not been strictly aligned with answering health policy and public health research questions, the depth and breadth of these data collection efforts around the world together with advances in data science open up exciting opportunities to answer important and timely health policy and public health questions that can help us improve health equity and population health.

## Supplementary Material

qxaf158_Supplementary_Data

## References

[1] Health research data for the world, UK Biobank . July 25, 2024. Accessed August 4, 2025. https://www.ukbiobank.ac.uk

[2] Denny J, Rutter J, Goldstein D, et al All of US research program investigators. The “all of US” research program. N Engl J Med. 2019;381(7):668–676. 10.1056/NEJMsr1809937 31412182 PMC8291101

[3] Baciu AB, Martinez RM. Revisiting the IOM reports and envisioning a promising future for public health. Am J Public Health. 2024;114(5):495–500. 10.2105/AJPH.2024.307584 38598765 PMC11008293

[4] Allen CG, Olstad DL, Kahkoska AR, et al Extending an antiracism lens to the implementation of precision public health interventions. Am J Public Health. 2023;113(11):1210–1218. 10.2105/AJPH.2023.307386 37651661 PMC10568499

[5] Livingston G, Huntley J, Liu KY, et al Dementia prevention, intervention, and care: 2024 report of the Lancet standing commission. Lancet Lond Engl. 2024;404(10452):572–628. 10.1016/S0140-6736(24)01296-0 39096926

[6] Basu S, Phillips RS, Berkowitz SA, Landon BE, Bitton A, Phillips RL. Estimated effect on life expectancy of alleviating primary care shortages in the United States. Ann Intern Med. 2021;174(7):920–926. 10.7326/M20-7381 33750188

[7] Kadakia KT, Desalvo KB. Transforming public health data systems to advance the population's health. Milbank Q. 2023;101(S1):674–699. 10.1111/1468-0009.12618 37096606 PMC10126962

[8] Abegaz TM, Ahmed M, Sherbeny F, Diaby V, Chi H, Ali AA. Application of machine learning algorithms to predict uncontrolled diabetes using the all of US research program data. Healthc Basel Switz. 2023;11(8):1138. 10.3390/healthcare11081138 PMC10137945 37107973

[9] Wang VHC, Cuevas AG, Osokpo OH, et al Discrimination in medical settings across populations: evidence from the all of US research program. Am J Prev Med. 2024;67:568–580. 10.1016/j.amepre.2024.05.018 38844146 PMC11819538

[10] Alvidrez J, Nápoles AM, Bernal G, et al Building the evidence base to inform planned intervention adaptations by practitioners serving health disparity populations. Am J Public Health. 2019;109(S1):S94–S101. 10.2105/AJPH.2018.304915 30699023 PMC6356120

[11] Boden-Albala B, Waddy SP, Appleton N, Kuczynski H, Nangle E, Parikh NS. Recruitment, inclusion, and diversity in clinical trials. In: The Science of Health Disparities Research: John Wiley & Sons, Ltd; 2021:413–428.

[12] Krieger N . Public health monitoring: an active phrase for vigilance, warning, guidance, and accountability. Am J Public Health. 2024;114(11):1199–1201. 10.2105/AJPH.2024.307850 39356996 PMC11447787

[13] Wang VHC, Holm J, Pagán JA. Use of calibration to improve the precision of estimates obtained from All of Us data. J Am Med Inform Assoc. 2024;31:2985–2988. 10.1093/jamia/ocae181 38981110 PMC11631143

[14] Hodge JG, Piatt JL, White EN, Gostin LO. Public health legal protections in an era of artificial intelligence. Am J Public Health. 2024;114(6):559–563. 10.2105/AJPH.2024.307619 38635946 PMC11079834

[15] Wang VHC, Lei J, Shi T, Pagán JA. Weighting the United States All of Us research program data to known population estimates using raking. Prev Med Rep. 2024;43:102795. 10.1016/j.pmedr.2024.102795 39026566 PMC11257137

